# Phenotypic Selection on Flower Color and Floral Display Size by Three Bee Species

**DOI:** 10.3389/fpls.2020.587528

**Published:** 2021-01-14

**Authors:** Johanne Brunet, Andrew J. Flick, Austin A. Bauer

**Affiliations:** ^1^United States Department of Agriculture, Agricultural Research Service, Madison, WI, United States; ^2^Agricultural Research Service Research Participation Program – Oak Ridge Institute for Science and Education, Madison, WI, United States; ^3^Department of Entomology, University of Wisconsin, Madison, WI, United States

**Keywords:** bumble bee, correlational selection, floral display size, flower color, honeybee, leafcutting bee, phenotypic selection

## Abstract

Plants exhibit a wide array of floral forms and pollinators can act as agent of selection on floral traits. Two trends have emerged from recent reviews of pollinator-mediated selection in plants. First, pollinator-mediated selection on plant-level attractants such as floral display size is stronger than on flower-level attractant such as flower color. Second, when comparing plant species, distinct pollinators can exert different selection patterns on floral traits. In addition, many plant species are visited by a diverse array of pollinators but very few studies have examined selection by distinct pollinators. In the current study, we examined phenotypic selection on flower color and floral display size by three distinct bee species, the European honey bee, *Apis mellifera*, the common eastern bumble bee, *Bombus impatiens*, and the alfalfa leafcutting bee, *Megachile rotundata*, foraging on *Medicago sativa.* To estimate phenotypic selection by each bee species and for all bees combined simultaneously and on the same group of plants, we introduce a new method that combines pollinator visitation data to seed set and floral trait measurements data typical of phenotypic selection study. When comparing floral traits, all bee species selected on the number of racemes per stem and the number of stems per plant, two components of floral display size. However, only leafcutting bees selected on hue or flower color and only bumble bees selected on chroma or darkness of flowers. Selection on chroma occurred via correlational selection between chroma and number of open flowers per raceme and we examine how correlational selection may facilitate the evolution of flower color in plant populations. When comparing bee species, the three bee species exerted similar selection pattern on some floral traits but different patterns on other floral traits and differences in selection patterns were observed between flower-level and plant-level attractants. The trends detected were consistent with previous studies and we advocate the approach introduced here for future studies examining the impact of distinct pollinators on floral trait evolution.

## Introduction

Plants exhibit a high level of floral trait diversity. Flower size, flower color, flower shape, and various aspects of floral display size can vary among plants in a population, among populations of a plant species or among plant species (Brunet, 2009; Dart et al., 2012). The role of pollinators in shaping such floral diversity has been of great interest to evolutionary biologists (Galen, 1996; Fishman and Willis, 2008; Harder and Johnson, 2009; Sletvold et al., 2017). In the last three decades, the attention has focused on identifying the role of pollinators, as opposed to other biotic or to abiotic factors, as agent of selection on floral traits (Strauss and Whittall, 2006; Parachnowitsch and Kessler, 2010; Caruso et al., 2019). The two literature reviews of phenotypic selection in plants have indicated that selection on floral traits by pollinators tend to be greater than by herbivores (Parachnowitsch and Kessler, 2010; Caruso et al., 2019) but can be of similar strength as selection by abiotic factors (Caruso et al., 2019).

To isolate the impact of pollinators on selection of floral traits, it has been suggested to measure phenotypic selection in two groups of plants, one group of hand-pollinated and one group of open-pollinated plants (Fishman and Willis, 2008; Sandring and Ågren, 2009; Parachnowitsch and Kessler, 2010; Sletvold et al., 2017). Selection gradients are estimated for each group and the difference in the selection gradients between the hand-pollinated and the open-pollinated treatments is attributed to pollinator-mediated selection on the floral traits of interest. When concentrating on directional selection for studies that compared hand-pollinated and open-pollinated treatments, two patterns emerged. First, pollinators differentially selected on distinct categories of floral traits. Selection was strongest on floral traits associated with pollinator efficiency such as the length of the corolla tube, followed by plant level traits associated with pollinator attraction such as floral display size and finally selection was weakest for flower level traits associated with pollinator attraction such as flower size and flower color (Caruso et al., 2019). Second, distinct pollinators had different impacts on the selection of floral traits and, among plant species, long-tongue flies or birds tended to exert the strongest selection on floral traits and Lepidoptera the weakest (Caruso et al., 2019).

Few studies have compared selection by distinct pollinators within a plant species (Sahli and Conner, 2011; Kulbaba and Worley, 2012, 2013). Conflicting selection among pollinators was identified for some floral traits while for other traits distinct pollinators exerted similar patterns of selection (Sahli and Conner, 2011). For example, in *Polemonium brandegeei*, hummingbirds selected for stigmas exserted beyond the anthers and for longer and wider corolla tubes while hawkmoths selected for stigmas recessed below the anthers and for narrower corolla tubes (Kulbaba and Worley, 2012, 2013). These studies examined each pollinator separately and on different sets of plants (Kulbaba and Worley, 2013) or examined pollinators separately and combined in cages (Sahli and Conner, 2011). But plants in natural populations are differentially visited by distinct pollinators whose abundance and efficiency vary and it would be useful to quantify the impact of the major pollinators on the floral traits of interest simultaneously and on the same group of plants.

Pollinator visitation has been used as a proxy for reproductive success in some phenotypic selection studies (Campbell et al., 1997; Zhao and Wang, 2015). Here, we propose to combine pollinator observations with measurements of seed set and floral traits of plants to examine phenotypic selection on floral traits by distinct pollinators. Each plant in the population is expected to receive differential visits by the distinct pollinator species and a different proportion of its flowers will be visited by each species. Such proportions can be used to differentially attribute seeds set on a plant to the distinct pollinators. In addition, data on pollinator efficiency can be combined with flower visits data to proportionally attribute seeds to each pollinator species. Relative fitness (RF) and phenotypic selection can then be measured within each bee species. Phenotypic selection by all pollinators combined can be measured using total seed set per plant to calculate RF. This approach is developed here to illustrate how phenotypic selection by all pollinators combined can be differentially attributed to each pollinator species as we study phenotypic selection on flower color and floral display size by three bee species in *Medicago sativa*. We determine whether selection by pollinators is stronger for plant-level attractants like floral display size than for flower-level attractants such as flower color (Caruso et al., 2019). We also examine whether the three bee species exert similar or different patterns of selection on these floral traits and how this translates into the overall pattern of selection on the plants. Measuring selection on floral traits by different bee species on the same group of plants provides a more realistic depiction of pollinator-mediated selection on floral traits in plant populations.

## Materials and Methods

### Study Species

*Medicago sativa* L. is an open-pollinated perennial legume that requires bees for seed production. Flowers are clustered into racemes and plants exhibit variation in the number of open flowers per raceme, number of racemes per stem (inflorescence), and number of stems per plant and flower color can also vary, ranging from shades of purple, to white, to yellow (Bauer et al., 2017).

*Medicago sativa* flowers require tripping for pollination, where pollinators apply pressure to the keel of the flowers which releases its anthers and stigmas. Flowers remain open following tripping but there is little evidence of further pollen deposition by pollinators on already tripped flowers (J. Brunet, pers. obs.). The tripping rate, the proportion of visited flowers that are tripped by a pollinator, varies among bee species (Cane, 2009; Brunet and Stewart, 2010; Pitts-Singer and Cane, 2011). Typically, alfalfa leafcutting bees have the highest tripping rate, followed by bumble bees and finally honey bees (Pitts-Singer and Cane, 2011; Brunet et al., 2019). Honey bees (*Apis mellifera*) and alfalfa leafcutting bees (*Megachile rotundata*) are used as managed pollinators in alfalfa seed production fields. In addition, many wild bee species, including the common eastern bumble bee (*Bombus impatiens*), are known to visit and effectively pollinate alfalfa (Brookes et al., 1994; Brunet and Stewart, 2010).

### Experimental Set Up

Five patches of *M. sativa* with 81 plants per patch, initially planted 0.3 m apart, were set up in a linear arrangement at the West Madison Agricultural Research Station in Madison, WI. One bumble bee hive was set up at the center edge, one honey bee hive 30 m away, and a leafcutting bee domicile was set up at the northwest corner facing southeast. About 1.2 lbs of leafcutting bees were released prior to *M. sativa* peak bloom. Floral trait and fitness measurements were obtained from all flowering plants in two center patches where each plant was numbered.

### Floral Traits

The floral traits examined in this study included components of floral display size and flower color. For floral display size, we recorded the number of stems per plant, racemes per stem and open flowers per raceme. For each flowering plant, we counted the number of stems and the number of racemes per stem on ten randomly selected stems or on all stems if a plant had fewer than ten stems. The number of open flowers per raceme was recorded on ten randomly selected racemes per plant. The average number of racemes per stem and open flowers per raceme were tabulated for each plant.

Flower color was determined from spectral measurements of the banner petal for three flowers per plant using the USB 4000 spectrophotometer (Ocean Optics, Orlando, Fl., 350–1,000 nm). Reflectance data were analyzed using Spectra Suite v.10.7.1 software (Ocean Optics). Flowers of *M. sativa* do not reflect in the UV range (Bauer et al., 2017) and spectral measurements were taken in the visible light range (400–700 nm). We used equations from Endler (1990) as modified by Smith (2014) to calculate three components of flower color: chroma (darkness or saturation), hue (color), and reflectivity (brightness). Details of these calculations can be found in Bauer et al. (2017). A plant value represented the average of the three flowers.

An alternative would have been to use a hexagon color vision model, a method that considers bee photoreceptors when quantifying color (Chittka, 1992; Chittka and Kevan, 2005). We have used such models to examine how flower color affected the choice of plants by bees (Bauer et al., 2017). However, in this study, while bees may be doing the selection, they are selecting on the plant traits and not on their perception of those traits. We thus chose hue, chroma and receptivity to describe flower color. While the best method to quantify flower color when pollinators are selecting on the trait may deserve further attention, such discussion is beyond the scope of this study.

### Female Reproductive Success

We used the total number of seeds produced per plant as a measure of female reproductive success. On each plant, on ten randomly selected stems or all stems if a plant had fewer than ten stems, we counted the number of pods per stem. A pod is a fruit developing from one flower on a raceme. We collected ten randomly selected fruiting racemes per plant and placed each one in an individually marked paper coin envelope. In the laboratory, the number of pods per raceme were recorded and pods were shredded to obtain the number of seeds per raceme. For each plant, we obtained the average number of mature seeds per pod per raceme and, using the 10 fruiting racemes per plant, we calculated the average number of mature seeds per pod on a plant. To obtain the total number of seeds set per plant, we multiplied the average number of pods per stem by the average number of seeds per pod and multiplied this value by the number of stems produced on a plant.

#### Proportion of Seeds Attributable to Each Bee Species

To estimate the proportion of seeds on each plant attributable to a given bee species, we used available data on the number of flower visits to a plant by each of the three bee species. Pollinator visitation data were collected on these plants during a two-week observation period at peak bloom for *M. sativa* the year of the study (Bauer et al., 2017). To determine the number of pollinator visits to a plant, we followed bees in a patch and two observers recorded each plant visited by a bee, the number of racemes visited on a plant and flowers visited per raceme on each plant until the bee left the patch or was lost to the observers. This provided floral visits by at least one of the three pollinators for most plants in the two patches (Supplementary Data). The pattern of visitation in the patches was typical for the major bee species visiting *M. sativa* throughout its flowering period. To attribute the number of seeds to a bee species based on the number of flower visits, for each plant, we multiplied the proportion of flowers visited by each of the three bee species by the number of seeds set on that plant. This approach links, for each plant, the pollinator visitation data to its seed set during that period, as seeds were collected about four weeks following pollinator observations, the period it takes for fruits and seeds to mature in this plant species.

The number of flowers visited by a bee species is a useful measure of pollinator visits, but to better link floral visits to seed set we also integrated the tripping rate of a bee species to the floral visitation data. In *M. sativa*, flowers must be tripped before they can produce seeds and tripping rate varies among bee species (Cane, 2009; Pitts-Singer and Cane, 2011). We obtained a second measure of pollinator visits which combined floral visits with the tripping rate of a bee species. Previous observations in the area indicated a tripping rate of 55% for bumble bee, 25% for honey bee (Brunet and Stewart, 2010) and 80% for leafcutting bee under warm temperatures typical of alfalfa seed-production fields (Brunet et al., 2019). For each plant, the number of flowers visited by a bee species on that plant was multiplied by the bee species specific tripping rate. We call this measure the number of flowers tripped by a bee species. For each plant, we calculated the number of tripped flowers by each bee species and the proportion of flowers tripped by each bee species. We multiplied these proportions by the number of seeds set on the plant to assign seeds to each of the three bee species based on the number of tripped flowers.

### Plant Relative Fitness

Plant relative fitness (RF) was estimated by dividing the absolute fitness of a plant by the mean absolute fitness of the group of plants under consideration (Lande and Arnold, 1983). The absolute fitness of a plant was quantified as the number of seeds set on a plant. RF was obtained for all plants for which floral trait measurements were available (*N* = 153). We calculated RF of a plant over all bees, based on the total number of seeds it produced, and within each bee species. Within a bee species, RF was the number of seeds on a plant attributable to a given bee species, based either on the proportion of flowers visited or the proportion of flowers tripped by a specific bee species, divided by the mean for that bee species. Using this approach, the mean RF was 1.0 within each bee species and potential differences in seed production across pollinators were removed. We also calculated the opportunity for selection for overall RF and for RF by bee species based on proportion of visits or proportion of tripped flowers. Opportunity for selection was measured as the variance in RF.

### Phenotypic Selection

To measure phenotypic selection, we examined the relationship between the trait value of a plant and its RF (Lande and Arnold, 1983). Each floral trait examined was scaled such that its mean was 0 and its variance was 1: (trait value – trait mean)/trait standard deviation. We performed phenotypic selection analyses on the number of stems per plant, the number of racemes per stem, the number of open flowers per raceme, and hue, chroma and reflectivity. We first performed phenotypic selection analyses using RF calculated over the total seed set of a plant. We also examined phenotypic selection within each bee species, where RF was calculated as explained earlier, either based on proportion of flowers visited or proportion of flowers tripped by a bee species on each plant. RF was relativized and traits standardized within each bee species which eliminated any potential differences in traits or fitness across bee species. The number of plants was similar for overall fitness and within each bee species and represented plants with floral traits and seed set data (*N* = 153). The number of plants that received no visits by a specific bee species did vary, with a greater number of plants not visited by leafcutting bee (*N* = 107 plants), followed by honey bee (*N* = 46) and last bumble bee (*N* = 16).

We used regression analyses, examining linear and non-linear regressions, to estimate various selection parameters, following the methods suggested by Lande and Arnold (1983). Untransformed variables were used to obtain the values of the selection coefficients. To obtain the statistical significance of the selection coefficients, RF values were log transformed in order to improve the model’s residuals. This procedure was followed because selection coefficients are not known to be affected by a poorly fit model while the probability values are (Lande and Arnold, 1983; Mitchell-Olds and Shaw, 1987; Brodie and Janzen, 1996). In addition, due to the large number of zeros, the model’s residuals for leafcutting bee still indicated a poor fit to the data after transforming RF. We therefore used bootstrapping to estimate the 95% confidence intervals around the selection coefficients and determine whether they were statistically significant (Davison and Hinkley, 1997). We used bootstrapping for all cases for comparison purpose. We performed 1,000 bootstraps using the bootstrap function in the package “boot” (Canty and Ripley, 2020) in R (version 3.6.1).

For directional selection, we estimated the selection differential (S*_*i*_*), which represents the change in the population mean of trait (*i*) after selection (Arnold and Wade, 1984). The selection differential can be obtained from the slope of a linear regression between the standardized value of a trait and the corresponding plant RF. This coefficient includes both direct and indirect selection and multiple regression analyses were performed to isolate direct selection. The partial regression coefficient for a trait represents the selection gradient (β*i*) for that trait (*i*) and illustrates direct selection on a trait after removing indirect selection from all other traits present in the analysis. When traits are correlated, a trait that appears to respond to selection may simply be correlated to the trait under selection, hence the need to isolate direct selection. The coefficients S or β both represent directional selection and a positive value indicates that the phenotypic mean of a trait (*i*) increases under selection while it decreases when values of S*i* or β*i* are negative.

Because selection can also be non-linear and work on the shape of the trait distribution, we first added a quadratic term to the single regression and obtained the non-linear (quadratic) selection differential C_*ii*_ (Table 1), where C_22_ illustrates the non-linear (quadratic) term of the single regression. We then performed multiple regressions with linear, quadratic and cross product terms to obtain the non-linear or quadratic selection gradient γ_*ii*_, represented by the partial regression coefficient for the quadratic term, and to detect correlational selection γ_*ij*_ using the partial regression coefficient for the cross product terms (Table 1; Brodie, 1992; Roff and Fairbairn, 2012). The quadratic coefficient gradients were estimated as double the quadratic regression coefficients (Stinchcombe et al., 2008; Sahli and Conner, 2011).

**TABLE 1:** Selection parameters obtained based on different regression analyses using plant relative fitness and standardized floral traits.

Model	Single regression		Multiple regression	
Linear	S	Selection differential; slope; both direct and indirect selection	β	Selection gradient; partial regression coefficient; direct selection
Non-linear (linear and quadratic terms)	C_*ii*_	Non-linear or quadratic selection differential is C_22_		
Non-linear (linear, quadratic and cross product terms)			γ_*ii*_	Non-linear or quadratic selection gradient; partial regression coefficient of quadratic term
Non-linear (linear, quadratic and cross product terms)			γ_*ij*_	Correlational selection gradient; Partial regression coefficient of cross product term

*The subscript i indicates trait(i) and j indicates a separate trait.*

We graphically illustrated the statistically significant cross product terms representing correlational selection gradients using the function “persp” in R (R Core Team, 2019). To represent the non-linear selection for the statistically significant quadratic selection gradients we used generalized additive models (GAMs) using the “mgcv” package in R (Wood, 2011). These models automatically fit a spline regression (Wood, 2011). Results from the GAMs were plotted using “ggplot2”(Wickham, 2016) and “gridExtra”(Auguie, 2017).

Besides using regression analyses, we also examined the distributional selection gradient on the floral traits (Henshaw and Zemel, 2017). This measures total selection on a trait and can be broken down into a directional component (dD) illustrating selection on a trait mean and a non-directional component (dN) that reflects selection on the shape of the trait distribution. This approach permits estimation of the general selection differential (S) and selection gradients (β). We used the R code available from Github^1^ to run distributional selection differential analyses on our data following Henshaw and Zemel, 2017.

## Results

### Floral Traits, Bee Visits, and Plant Relative Fitness

Data on floral traits and seed set were recorded for 153 plants. We observed 8,727 flower visits on these plants, with 4,570 (52.3%) visits by bumble bees, 3,925 (45.0%) by honey bees and 232 (2.7%) flower visits by leafcutting bees. The average seed set for the 153 plants in this study was (mean ± STD) 754.67 ± 986.90, with a range from 6.63 to 9,874.62 seeds per plant. The plants in the experiment had (mean ± STD) 4.93 ± 3.41 racemes per stem with a range between 0.11 and 14.6 racemes per plant. Each value for a plant represents the average of ten readings per plant. Plants had 7.53 ± 2.44 open flowers per raceme with a range from 3 to 14.4 open flowers per raceme and 30.65 ± 16.4 stems per plant with a range between 6 and 87 stems. Flower color varied with (mean ± STD) chroma values of 1.29 ± 0.76 with a range from 0.257 to 3.48; reflectivity values of 3.76 ± 1.11 and a range from 1.76 to 7.14 and hue values of -0.012 ± 0.476 and a range from -0.802 to 1.21.

The opportunity for selection was 1.74 for overall fitness, calculated using total seed set per plant. When RF was based on the proportion of flower visits, the opportunity for selection was 1.24 for bumble bee, 2.53 for honey bee and 46.48 for leafcutting bee. The average seed set attributable to each bee species, based on proportion of flower visits, was 396.27 seeds per plant for bumble bee; 261.03 for honey bee; and 61.68 for leafcutting bee. When RF was based on the proportion of tripped flowers, the opportunity for selection was 1.15 for bumble bee, 3.16 for honey bee and 35.54 for leafcutting bee. The average seed set of a plant attributable to each bee species was 453.32 seeds for bumble bee, 187.24 seeds for honey bee, and 84.25 seeds for leafcutting bee.

### Phenotypic Selection

#### All Bees Combined

**FIGURE 1:**
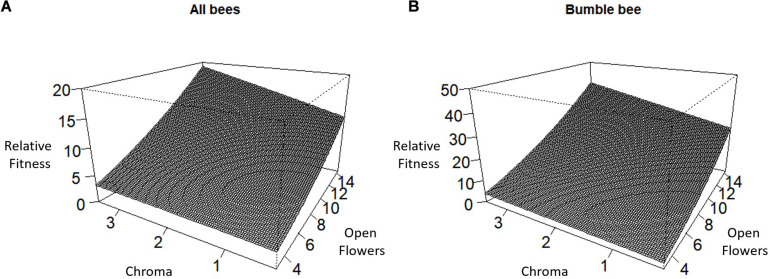
Correlational selection between the number of open flowers per raceme and flower chroma or darkness for **(A)** All bees combined and **(B)** Bumble bee.

Over all pollinators combined, we observed a positive directional selection differential *S* and selection gradient β on the number of stems per plant indicating selection to increase the number of stems per plant (Table 2). For the number of racemes per stem, there was a statistically significant positive directional selection differential *S* and selection gradient β indicative of selection for an increase in the number of racemes. However, we also detected a statistically significant negative quadratic selection differential *C*_22_ although the quadratic selection gradient γ*_*ii*_* was not statistically significant, suggesting indirect non-linear selection on the number of racemes per stem (Table 2). Finally, there was a statistically significant positive correlational selection between the number of open flowers per raceme and the chroma or darkness of flowers (γ_*FlrChr*_), at least for the regression model with log transformed RF (Table 2 and Figure 1A). Under correlational selection, particular combinations of two traits expressed together in the same individual are favored and here pollinators favored plants with more open flowers per raceme and darker flowers.

**TABLE 2:** Phenotypic selection with relative fitness calculated over all bees (*N* = 153) using either the regression model with log transformed relative fitness or bootstrapping to determine the statistical significance of the selection coefficients.

Statistical Method		Log transformed		Bootstrap		
Trait	Selection coefficient	Estimate	*P*	Estimate	Lower 95%	Upper 95%
Racemes per Stem	S	0.297	<0.0001	0.297	0.196	0.422
Racemes per Stem	β	0.199	<0.0001	0.199	0.106	0.308
Racemes per Stem	C_22_	−0.139	0.004	−0.139	−0.272	−0.055
Stems per plant	S	0.612	<0.0001	0.612	0.419	0.801
Stems per plant	β	0.578	<0.0001	0.578	0.383	0.764
Open Flowers per raceme	γ_*FlrChr*_	0.370	0.022	0.370	NS	NS

*The methodology followed to obtain the different selection coefficients is summarized in Table 1. The letter P stands for probability and NS for not statistically significant. Flr stands for open flowers per raceme and Chr for flower chroma.*

#### Bumble Bee

For bumble bees, the positive directional selection differential *S* and gradient β were both statistically significant for the number of racemes per stem and for the number of stems per plant suggesting selection to increase both traits (Table 3). In addition, we observed a statistically significant positive correlational selection gradient between the number of open flowers on a raceme and the darkness of a flower (chroma) (γ_*FlrChr*_) for all cases except when RF was based on the number of tripped flowers and the statistical significance of selection coefficients were tested using bootstrapping (Table 3). Bumble bees favored plants with more open flowers per raceme and with darker flowers (Figure 1B). There was also a positive correlational selection gradient between the number of open flowers and flower reflectivity (γ_*FlrRef*_) but it was only statistically significant when RF was based on the proportion of flowers visited and when log transformed RF regression model was used to detect the significance of the selection coefficients (Table 3).

**TABLE 3:** Phenotypic selection for bumble bee with relative fitness calculated based either on the proportion of flowers visited or of flowers tripped by bumble bees.

N = 153		Proportion of flowers visited					Proportion of tripped flowers				
		Log transformed		Bootstrap			Log transformed		Bootstrap		
Trait	Selection coefficient	Estimate	*P*	Estimate	Lower 95%	Upper 95%	Estimate	*P*	Estimate	Lower 95%	Upper 95%
Racemes per Stem	S	0.276	<0.0001	0.276	0.137	0.423	0.283	<0.0001	0.283	0.157	0.420
Racemes per Stem	β	0.223	0.006	0.223	0.053	0.405	0.212	<0.0001	0.212	0.070	0.371
Stems per plant	S	0.434	<0.0001	0.434	0.190	0.733	0.475	<0.0001	0.475	0.226	0.769
Stems per plant	β	0.396	<0.0001	0.396	0.146	0.703	0.437	<0.0001	0.437	0.183	0.731
Open Flowers per raceme	γ_*FlrChr*_	0.706	0.011	0.706	0.008	1.24	0.604	0.018	0.604	NS	NS
Open Flowers per raceme	γ_*FlrRef*_	0.588	0.032	0.588	NS	NS	0.115	NS	0.115	NS	NS

*The statistical significance of the selection coefficients was determined using either regression models with log transformed relative fitness or using bootstrapping. The methodology followed to obtain the different selection coefficients is summarized in Table 1. The letter P stands for probability and NS for not statistically significant. Flr stands for open flowers per raceme, Chr for flower chroma and Ref for flower reflectivity.*

#### Honey Bee

**FIGURE 2:**
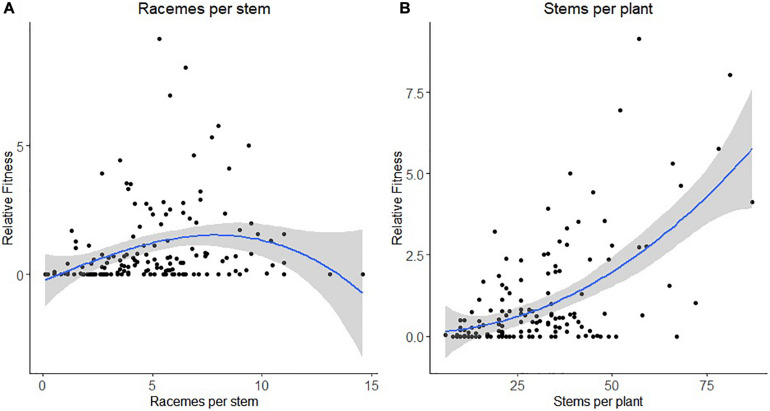
Non-linear selection by honey bee for panel **(A)** the number of racemes per stem and **(B)** the number of stems per plant. The blue line represents the predictive spline curve while the gray area encompasses the 95% confidence intervals.

For honey bee, there was a statistically significant negative quadratic selection differential (C_22_) and quadratic selection gradient (γ*_*ii*_*_)_ for the number of racemes per stem indicative of non-linear selection (Table 4). Results of the spline regression analysis indicates that honey bees exert some stabilizing selection on the number of racemes per stem (Figure 2A). For the number of stems per plant, both the positive directional selection differential *S* and selection gradients β were statistically significant but we also detected non-linear positive selection, suggestive of disruptive selection, with statistically significant quadratic selection differential (C_22_) and gradient (γ*_*ii*_*) but only when bootstrapping was used to determine the statistical significance of the selection coefficients (Table 4). The coefficient of directional selection was much larger than the non-linear selection coefficient (Table 4) which translated into mostly directional selection for increased number of stems per plant as indicated by the spline regression analysis (Figure 2B). Patterns were similar whether RF was based on the proportion of visited or tripped flowers (Table 4).

**TABLE 4:** Phenotypic selection for honey bee with relative fitness calculated using either the proportion of flowers visited or the proportion of flowers tripped by honey bees.

N = 153		Proportion of flowers visited					Proportion of flowers tripped				
		Log transformed		Bootstrap			Log transformed		Bootstrap		
Trait	Selection coefficient	Estimate	*P*	Estimate	Lower 95%	Upper 95%	Estimate	*P*	Estimate	Lower 95%	Upper 95%
Racemes per Stem	S	0.314	0.004	0.314	0.108	0.548	0.292	0.011	0.292	0.084	0.579
Racemes per Stem	C_22_	−0.268	<0.0001	−0.268	−0.443	−0.157	−0.256	0.002	−0.256	−0.455	−0.132
Racemes per Stem	γ_*rcpsrcps*_	−0.234	0.01	−0.234	−0.450	−0.057	−0.223	0.026	−0.223	−0.465	−0.009
Stems per plant	S	0.907	<0.0001	0.907	0.605	1.262	0.951	<0.0001	0.951	0.649	1.379
Stems per plant	β	0.890	<0.0001	0.890	0.580	1.273	0.947	<0.0001	0.745	0.489	1.174
Stems per plant	C_22_	0.186	NS	0.186	0.012	0.400	0.196	NS	0.196	0.017	0.349
Stems per plant	γ_*stppstpp*_	0.230	NS	0.230	0.025	0.536	0.246	NS	0.246	0.028	0.598

*The statistical significance of the selection coefficients was determined using either regression models with log transformed relative fitness or using bootstrapping. The methodology used to obtain the different selection coefficients is summarized in Table 1. The letter P stands for probability and NS for not statistically significant. Rcps stands for racemes per stem and stpp stands for stems per plant.*

#### Leafcutting Bee

**FIGURE 3:**
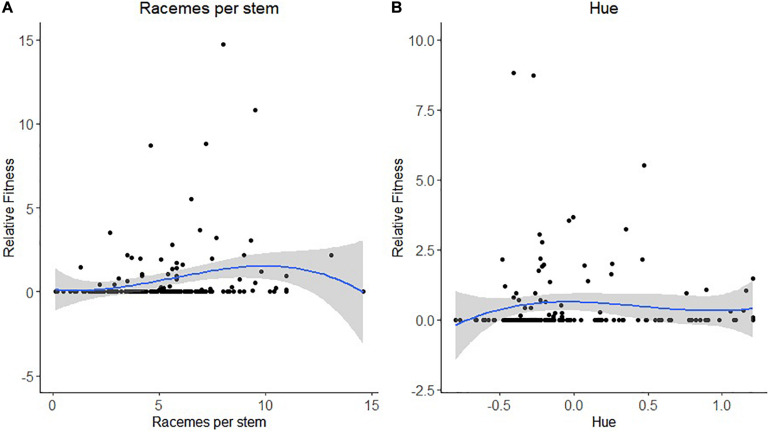
Non-linear selection by leafcutting bees for **(A)** the number of racemes per stem and **(B)** flower hue or color. The blue line represents the predictive spline curve while the gray area encompasses the 95% confidence intervals.

For leafcutting bee, only bootstrapping was used to determine the statistical significance of the selection coefficients. For the number of racemes per stem, we detected both directional and non-linear selection (Table 5). There was a statistically significant positive selection differential *S* and gradient β but also a statistically significant negative quadratic selection differential C_22_ and gradient γ*_*ii*_* at least when RF was based on the proportion of visited flowers (Table 5). The spline analysis indicated that leafcutting bees exerted some stabilizing selection on the number of racemes per plant (Figure 3A). For the number of stems per plant, we detected a positive directional selection differential *S* and gradient β favoring plants with more stems. Finally, we observed a statistically significant negative quadratic selection gradient γ*_*ii*_* for flower color or hue, indicating some stabilizing selection on hue by leafcutting bees (Table 5 and Figure 3B).

**TABLE 5:** Phenotypic selection for leafcutting bee with relative fitness calculated using either the proportion of flowers visited or the proportion of flowers tripped by leafcutting bees.

N = 153		Proportion of flowers visited			Proportion of flowers tripped		
Trait	Selection coefficient	Estimate	Lower 95%	Upper 95%	Estimate	Lower 95%	Upper 95%
Racemes per Stem	S	0.512	0.167	1.231	0.501	0.159	1.090
Racemes per Stem	β	0.426	0.117	1.433	0.387	0.108	1.024
Racemes per Stem	C_22_	−0.235	−0.631	−0.042	−0.226	−0.532	−0.033
Racemes per Stem	γ_*rcpsrcps*_	−0.249	−1.007	−0.001	−0.249	NS	NS
Stems per plant	S	0.724	0.314	1.585	0.791	0.338	1.950
Stems per plant	β	0.628	0.216	1.435	0.696	0.244	1.703
Hue	γ_*huehue*_	−0.620	−1.940	−0.068	−0.628	−1.810	−0.063

*The statistical significance of the selection coefficients was only determined using bootstrapping due to the high number of plants with no leafcutting bee visits. The methodology used to obtain the different selection coefficients is summarized in Table 1. The abbreviation NS stands for not statistically significant. Rcps stands for racemes per stem and hue for flower hue.*

### Distributional Selection Differential

When performing distributional selection differential (DSD) analyses, we detected positive directional selection for the number of racemes per stem and the number of stems per plant for all bees combined and for each bee species (Table 6). We did not detect non-linear selection on any components of floral display size and did not detect selection on any components of flower color for either all bees combined or any of the bee species (Table 6). We present the DSD results to contrast with the results obtained using the Lande and Arnold (1983) approach. We will leave other studies to discuss discrepancies between approaches and below concentrate on the results obtained using the more traditional method originally proposed by Lande and Arnold (1983).

**TABLE 6:** Distributional selection differential on the floral traits.

Floral Traits	DSD	Probability	dD	Probability	dN	Probability	S	Probability	delta δ	Beta β
**All bees**										
Racemes per stem	**0.333**	**0.002**	**0.328**	**0.002**	0.006	0.878	**0.328**	**0.002**	0.278	0.193
Stems per plant	**0.806**	**0.000**	**0.806**	**0.000**	0.000	0.984	**0.806**	**0.000**	0.732	0.786
Total flowers	0.060	0.973	0.017	0.863	0.043	0.398	0.017	0.863	0.615	−0.031
Hue	0.064	0.911	0.006	0.955	0.058	0.173	0.006	0.955	0.492	0.016
Chroma	0.050	0.990	0.010	0.931	0.040	0.353	−0.010	0.931	0.241	−0.115
Reflectivity	0.088	0.774	0.040	0.709	0.047	0.345	0.040	0.709	0.214	0.038
**Bumble bee**										
Racemes per stem	**0.276**	**0.002**	**0.276**	**0.002**	0.000	0.983	**0.276**	**0.002**	0.178	0.223
Stems per plant	**0.439**	**0.000**	**0.434**	**0.000**	0.005	0.871	**0.434**	**0.000**	0.399	0.396
Total flowers	0.064	0.904	0.057	0.538	0.008	0.825	0.057	0.538	0.120	0.008
Hue	0.061	0.881	0.010	0.915	0.051	0.194	0.010	0.915	1.463	0.120
Chroma	0.065	0.842	0.038	0.679	0.027	0.474	0.038	0.679	−0.063	−0.119
Reflectivity	0.116	0.347	0.086	0.346	0.030	0.504	−0.086	0.346	0.149	−0.184
**Honey bee**										
Racemes per stem	**0.370**	**0.004**	**0.314**	**0.016**	0.055	0.429	**0.314**	**0.016**	0.229	0.132
Stems per plant	**0.907**	**0.000**	**0.907**	**0.000**	0.000	0.983	**0.907**	**0.000**	0.849	0.890
Total flower	0.156	0.408	0.142	0.275	0.014	0.790	0.142	0.275	0.111	0.065
Hue	0.159	0.339	0.118	0.367	0.041	0.452	0.118	0.367	0.239	0.140
Chroma	0.116	0.642	0.111	0.395	0.006	0.857	0.111	0.395	0.124	0.091
Reflectivity	0.093	0.901	0.061	0.634	0.032	0.618	0.061	0.634	0.336	0.122
**Leafcutting bee**										
Racemes per stem	**0.543**	**0.079**	**0.512**	**0.076**	0.031	0.800	**0.512**	**0.076**	0.507	0.426
Stems per plant	**0.734**	**0.016**	**0.724**	**0.015**	0.010	0.894	**0.724**	**0.015**	0.557	0.628
Total flowers	0.223	0.853	0.157	0.586	0.067	0.611	−0.157	0.586	0.313	−0.262
Hue	0.155	0.979	0.037	0.904	0.117	0.328	−0.037	0.904	2.529	0.204
Chroma	0.323	0.414	0.275	0.351	0.048	0.640	0.275	0.351	0.565	0.149
Reflectivity	0.179	0.967	0.131	0.656	0.048	0.703	−0.131	0.656	−0.181	−0.042

*Relative fitness was based on the proportion of floral visits. Total selection is measured by DSD while dD represents the directional and dN the non-directional component of selection. The general selection differential is S and β is the selection gradient. Statistically significant values are bolded.*

## Discussion

### Selection on Flower Color Relative to Floral Display Size

The number of stems per plant and the number of racemes per stem were selected by all three bee species. In contrast, only leafcutting bees favored intermediate hue and bumble bees exhibited correlational selection between open flowers per raceme and flower chroma. Components of floral display size, plant-level attractants, were selected more consistently relative to components of flower color, a flower-level attractant (sensu Caruso et al., 2019). This trend was observed under conditions favorable to detect phenotypic selection (Sahli and Conner, 2011). The plants used in this study exhibited a high level of phenotypic variation in both flower color and floral display size and the variation in flower color was greater than typically occurs in wild *M. sativa* populations. We also observed strong opportunity for selection overall and within each pollinator species.

The foraging behavior of pollinators may help explain the difference between selection on plant-level and flower-level attractants. Pollinators forage for rewards and their goal is to collect pollen and nectar to provide for their young and feed themselves. Components of floral display size such as the number of racemes per stem and the number of stems per plant are both indicative of the amount of resources available on a plant. Bumble bees can determine whether a flower offers pollen or not and can detect the number of pollen-producing flowers on a plant. They are attracted to inflorescences, a plant-level attractant, based on the number of pollen-producing flowers (Brunet et al., 2015). Similarly, bumble bees may be able to detect the number of nectar-producing flowers on inflorescences (Makino and Sakai, 2007). Bumble bees, on the other hand, cannot distinguish between flowers presenting distinct amount of pollen, a flower-level attractant, unless it is linked to another trait such as flower size or flower color (Brunet et al., 2015; Thairu and Brunet, 2015). Similarly, while bees have innate preferences for flower color (Simonds and Plowright, 2004; Raine and Chittka, 2007), they learn to associate a flower color with a reward and can switch their preference of flower color for the color providing the most reward (Ings et al., 2009; Thairu and Brunet, 2015). The fact that plant-level attractants such as floral display size directly advertise resource availability to pollinators may help explain why, relative to flower-level attractants, they are more likely to be selected by pollinators within plant populations.

An association between a reward and flower color is more likely to occur among plant populations or plant species of distinct colors rather than within a population where the association between a color and a reward can be broken down by recombination (Brunet et al., 2015). This may help explain why flower color polymorphisms are more common among than within plant populations (Narbona et al., 2018). Pollinators have been suggested as the selective agents responsible for flower color polymorphisms among populations (Streisfeld and Kohn, 2007) and in some cases the genes responsible for the change in flower color associated with each pollinator have been elucidated (Streisfeld et al., 2013). Similarly, the genetic basis of flower color differences has been elucidated for some plant species and shown to be responsible for the pollinator preference (Bradshaw and Schemske, 2003; Hoballah et al., 2007). However, it remains unclear whether the pollinator preference created the flower color diversification or whether the association between flower color and pollinator arose following the fixation of the flower color in the population or species due to a different factor.

Within plant populations, correlational selection between flower color and floral display size may facilitate the evolution of flower color via pollinators. Correlational selection, as was observed in the current study between number of open flowers per raceme and flower chroma, provides a mechanism to associate a flower-level attractant like flower color to a plant-level attractant that advertise resource availability to a pollinator. Moreover, correlational selection leads to the development of genetic correlations between traits (Roff and Fairbairn, 2012) and correlational selection between a flower color and floral display size has been shown to increase the frequency of a color morph within a population even in the absence of differences between color morphs in seedling germination or survival (Gomez, 2000). Correlational selection by pollinators, between flower color and a plant-level attractant, may facilitate the maintenance of flower color polymorphism within plant populations. The role or correlational selection in the evolution of flower color in plant populations deserves more attention.

### Distinct Pollinators and Selection of Floral Traits

Distinct pollinators can exert different or conflicting selection on floral traits (Galen, 1989; Sahli and Conner, 2011; Kulbaba and Worley, 2013) and in the current study, we found different patterns of selection on some floral traits by the distinct bee species. For example, bumble bees exerted positive directional selection on the number of racemes per stem and favored plants with more racemes per stem while both honey bees and leafcutting bees exerted non-linear selection in the form of stabilizing selection favoring an intermediate number of racemes per stem. Distinct pollinators may also select on distinct floral traits. The common eastern bumble bee was the only bee species favoring darker flowers, when associated with racemes with more open flowers. Bumble bees were associated with correlational selection between these two floral traits. Leafcutting bees favored intermediate flower hue and honey bees did not select on any components of flower color. Finally, distinct pollinators can select on some floral traits in a similar way as we observed here with all three bee species favoring plants with more stems.

While illustrating how distinct pollinators can differentially or similarly influence floral traits, this study also links the selection by the three pollinators to the overall selection on the floral traits. Overall, there is directional selection on the number of racemes per stem and stems per plant with indirect non-linear selection on racemes per stem. There is also correlational selection between number of open flowers per raceme and flower chroma. Clearly bumble bees are solely responsible for the correlational selection while all three bee species exert directional selection on the number of stems per plant. While both honey bees and leafcutting bees exert some stabilizing selection on the number of racemes per stem, the overall selection is mostly directional. Bumble bees were the most abundant pollinators and better trippers than honey bees, the second most frequent visitors. The differential influence of the three bee species on floral traits indicates that the overall pattern of selection in a population will vary with the abundance and efficiency of its pollinators. We therefore, expect temporal or spatial variation in pollinators (Brunet, 2009; Narbona et al., 2018) to influence the temporal or spatial pattern of selection on floral traits (Kelly, 1992; Siepielski et al., 2009, 2013; Narbona et al., 2018). However, environmental factors may also vary among populations or temporally within populations and affect floral trait evolution (Schemske and Bierzychudek, 2001; Strauss and Whittall, 2006; Caruso et al., 2019). Interestingly, yearly variation in abiotic factors can modify the pattern of correlational selection (Maad, 2000). Both pollinators and abiotic factors should be considered when examining phenotypic selection of floral traits over time or space (Narbona et al., 2018; Sletvold, 2019).

### RF and Phenotypic Selection Within Bee Species

The methodology introduced in this study permits evaluation of phenotypic selection by distinct pollinators simultaneously, using the same set of plants. It more realistically describes the process of pollinator-mediated selection in natural populations. Sample sizes remain the same, over all bees and within each bee species, although the proportion of flowers not visited by a bee species may vary among bee species. Of interest is the fact that pattern of selection obtained over combined pollinators could be explained by the patterns observed for each bee species. Moreover, some selection patterns were only significant at the level of a bee species. For example, selection on flower color by leafcutting bees was not expressed at the whole plant level, likely because leafcutting bees were not as common in the study and were responsible for a lesser proportion of the seeds produced by the plants in the population.

We assigned the selection patterns observed in this study to pollinators rather than to another biotic or to abiotic factors. This approach was followed because the number of pollinator visits increase seed set in this plant species (Bauer et al., 2017); *M. sativa* plants set few seeds in the absence of pollinators (Bohart, 1957); plants were grown in a common environment minimizing variation in resource availability; and herbivory was not observed. Gathering pollination data in phenotypic selection studies will provide useful information on pollinator-mediated selection by distinct pollinators. We will further argue in a separate manuscript that comparing selection gradients between hand-pollinated and open-pollinated plants may not be the most efficient method to assign selection to pollinators (Brunet, in preparation).

The approach introduced in this study relies on good quality pollinator data and a link between visitation and seed set. The pollinator visitation data should be representative of the plant species under study over its flowering season. The plants used to collect pollinator data should represent the variation in floral display size that occurs spatially and temporally in the population. If pollinator types vary throughout the day or the flowering season, one should sample to reflect such variation. To link floral visits to seed set, it is best to sample seeds on visited plants after a period that reflects the time it takes for seeds to reach maturity. Finally, while applied to female reproductive success, the methodology could be extended to male reproductive success. In this case the proportion of floral visits to plant(*i*) is used for proportional visits by the distinct pollinators as it reflects the pollen leaving plant(*i*). The total seeds assignable to plant(*i*), on plant (*i*) if selfing occurs and on other plants in the population, represent the seed set for male function for plant(*i*). Results of this study illustrate how the approach proposed can attribute overall phenotypic selection patterns to individual pollinators and we therefore advocate the approach introduced here for future studies examining the impact of distinct pollinators on floral trait evolution.

## Conclusion

The methodology introduced to isolate and combine the phenotypic selection patterns of distinct bee species on floral traits provides patterns of selection similar to what has been observed in previous studies. The selection patterns observed over all bees could be assigned to specific bee species. All three bee species selected for components of floral display sizes but not all bees favored components of flower color although the selection coefficients were strong. This difference between plant-level and flower-level attractants could be explained by the fact that floral display size but not flower color directly advertises resource availability to pollinators. Spatial and temporal variation in the abundance of the distinct pollinators is expected to affect patterns of selection of flower traits, particularly for traits differentially selected by the distinct pollinators. Correlational selection between floral display size, a plant-level attractant, and flower color, a flower-level attractant, is expected to facilitate the evolution of flower color by pollinators within plant populations. Studies of pollinator-mediated selection would benefit from combining data on pollinator visitation rates together with seed set and measurements of floral traits when examining the impact of distinct pollinators on floral trait evolution.

## Data Availability Statement

The original contributions presented in the study are included in the article/Supplementary Material, further inquiries can be directed to the corresponding author/s.

## Author Contributions

JB conceived the study and wrote the manuscript. AB collected the data and samples from the field, processed the samples, and did preliminary data analyses. AF performed the data analyses for the manuscript, prepared the Figures, and provided comments. All authors contributed to the article and approved the submitted version.

## Conflict of Interest

The authors declare that the research was conducted in the absence of any commercial or financial relationships that could be construed as a potential conflict of interest.

## Footnotes

1 https://github.com/yoavzemel/dsd
